# Monitoring of Low-Level Wind Shear by Ground-based 3D Lidar for Increased Flight Safety, Protection of Human Lives and Health

**DOI:** 10.3390/ijerph16224584

**Published:** 2019-11-19

**Authors:** Pavol Nechaj, Ladislav Gaál, Juraj Bartok, Olga Vorobyeva, Martin Gera, Miroslav Kelemen, Volodymyr Polishchuk

**Affiliations:** 1 MicroStep-MIS, Čavojského 1, 841 04 Bratislava, Slovak; pavol.nechaj@microstep-mis.com (P.N.); juraj.bartok@microstep-mis.sk (J.B.); olga.vorobyeva@microstep-mis.com (O.V.); 2 Department of Astronomy, Physics of the Earth, and Meteorology, Comenius University in Bratislava, Bratislava 4, 842 48 Mlynská dolina, Slovak; Martin.Gera@fmph.uniba.sk; 3 Faculty of Aeronautics, Technical University of Košice, Rampová 7, 041 21 Košice, Slovak; miroslav.kelemen@tuke.sk; 4 Faculty of Information Technologies, Uzhhorod National University, Narodna Square, 3, 88000 Uzhhorod, Ukraine; volodymyr.polishchuk@uzhnu.edu.ua

**Keywords:** flight safety, lidar, low-level wind shear, microburst, gust front, windborne aerosol particles, protection of lives, public health

## Abstract

Low-level wind shear, i.e., sudden changes in wind speed and/or wind direction up to altitudes of 1600 ft (500 m) above-ground is a hazardous meteorological phenomenon in aviation. It may radically change the aerodynamic circumstances of the flight, particularly during landing and take-off and consequently, it may threaten human lives and the health of passengers, people at the airport and its surrounding areas. The Bratislava Airport, the site of this case study, is one of the few airports worldwide and the first in Central Europe that is equipped with a Doppler lidar system, a perspective remote sensing tool for detecting low-level wind shear. The main objective of this paper was to assess the weather events collected over a period of one year with the occurrences of low-level wind shear situations, such as vertical discontinuities in the wind field, frontal passages and gust fronts to increase the level of flight safety and protect human lives and health. The lidar data were processed by a computer algorithm with the main focus on potential wind shear alerts and microburst alerts, guided by the recommendations of the International Civil Aviation Organisation. In parallel, the selected weather events were analyzed by the nearby located meteorological radar to utilize the strengths of both approaches. Additionally, an evaluation of the lidar capability to scan dynamics of aerosol content above the airport is presented.

## 1. Introduction: Lidars in Aviation

### 1.1. Motivation of the Paper

A meteorological lidar is a remote sensing instrument for observing the properties of the atmosphere that are particularly related to the motion of the air, such as vertical wind profile, low-level wind shear, turbulence or wake vortices. It works on a similar principle like the Doppler meteorological radar: both instruments emit electromagnetic pulses, detect the reflected pulses from the targets, and estimate radial wind velocities on the basis of the Doppler effect. The most important difference between the lidar and the radar lies in the wavelength of the electromagnetic signal determining the scale of the targets capable to reflect the emitted pulses. While the weather radar is aimed at detecting water droplets, ice particles or other form of hydrometeors, the lidar focuses on the signal backscattered from atmospheric pollutants (aerosol, dust, salt, etc.). Consequently, the wavelengths of radars range from 1 to 10 cm (microwaves), whereas lidars utilize wavelengths of 1−10 μm (the infrared spectrum). 

Weather lidars represent promising technology for the detection of low-level wind shear, which is a hazardous phenomenon for the aircraft during the critical phases of flight, i.e., landing or take-off. Abrupt changes in wind speed and/or wind direction may dramatically influence the aerodynamic circumstances of the flight and may threaten the safety of the aircraft. Pilots and air traffic controllers are trained to identify and avoid such events or if necessary, to manage the critical situations. 

In addition to onboard weather radars, low-level wind shear is generally monitored by ground-based alerting systems. Two major and mutually complementary systems are being used at the largest US airports: the Terminal Doppler Weather Radar (TDWR) network, which is a system of weather radars [1], and the Low-Level Windshear Alert System (LLWAS) system, which is comprised of several anemometers installed in the vicinity of runways [2]. Lidars, on the other hand, are ambitious complements to the traditional methods of detection of low-level wind shear, mostly for their compactness and ability to provide high-resolution scans, both from the temporal and spatial perspective. A further advantage of the lidar technology is that lidars are not strictly bound to the near-ground measurement as are anemometers, but they can carry out specialized tasks, such as scanning the glide paths of the landing aircraft. Moreover, a combination of the lidar and radar techniques can cover the entire spectrum of meteorological phenomena since lidar is designed to detect meteorological processes in dry conditions, whereas radar monitors hydrometeors, i.e., phenomena with a wet character. 

The Hong Kong Observatory started to operate a pulsed Doppler lidar at Hong Kong International Airport in August 2002. Their system for low-level wind shear monitoring and alerting that was launched in December 2005 was the first in the world to be used in aviation meteorology. Its basic concept lies in frequent monitoring of the glide path to determine changes in the headwind that directly affect the landing aircraft [3,4,5,6]. Weipert et al. [7] reported that a wind shear detection system consisting of an X-band radar and lidar was installed at the two largest airports in Germany (Munich and Frankfurt am Main) in 2013. The same lidar as at the Frankfurt am Main is being operated at other major airports of the world such as John F. Kennedy International Airport in New York, Atlanta Hartsfield, San Francisco International, Heathrow London, and the Narita and Haneda Airports in Tokyo [8]. 

The lidar-based detection of low-level wind shear seem to be the key topic of interest in aviation. Nevertheless, Doppler lidars have also become an essential source of information in other specific disciplines such as turbulence alerting and forecast, detection of wake vortices, 2D or 3D wind field retrieval, visibility and mixing height monitoring [4,6,9,10,11]. 

The current paper presents the first outcomes of a 4-year-long (2016–2020) scientific project, devoted to developing a system for low-level wind shear detection and alerting by means of a Doppler lidar (Windcube 200S, Leosphere, Saclay, Île-de-France, France). The instrument provides a unique insight into 3D wind flows in a topographically complex region around the Bratislava Airport. We predominantly focused on the analysis of weather events that are typical for the location of the lidar and at the same time, are accompanied by considerable low-level wind shear. Relevant situations include vertical discontinuities in the wind field, stable vertical vortices from the nearby mountain range, and frontal passages. The presented study is part of systematic scientific work and permanent international cooperation. Its aim was to demonstrate our developments towards a lidar-based automated system for wind shear detection and alerting for operational usage in the air traffic control, focusing mostly on weather events that may endanger human lives and the health of passengers as well as people at the airport and in its vicinity. We attempted to make use the synergies between the existing and the new remote detection instruments (radar and lidar, respectively) and tested a proposed algorithm to issue alerts in case of the presence of significant low-level wind shear. 

The structure of the paper is as follows: Section 1 introduces the motivation of the paper and in a wider sense, the motivation for using the lidar technology for the remote detection of severe meteorological phenomena, such as downbursts that are accompanied by low-level wind shear. Section 2 deals with the concept of wind shear and its detection in aviation. In addition, Section 2 presents the topographical/climatological settings of the Bratislava Airport, and gives a brief overview on the technical details of the two remote sensing instruments that monitor the air space above the target location. In Section 3, four selected weather events are described and analyzed and the performance of the proposed algorithm to issue wind shear alerts and/or microburst alerts is evaluated. Finally, in the last sections of the paper, different aspects of our algorithm are discussed, the most important achievements are summarized and some paths towards future goals are delineated. 

### 1.2. Extreme Meteorological Phenomena in Aviation 

Storms have always been considered one of the greatest threats to aviation. They are accompanied by the potentially dangerous wind shear and downburst phenomena, both related to sudden changes in wind speed and direction. They may result in difficulties in aircraft control and at worst, can lead to an aircraft crash. They have been the main cause of some major aircraft crashes, e.g., at John F. Kennedy International Airport in New York in 1975 (Eastern Air Lines, Flight 66, [12]) or at Dallas/Fort Worth International Airport in 1985 (Delta Air Lines, Flight 191, [13]). Therefore, air transport authorities (e.g., the International Civil Aviation Organisation, ICAO) have been trying to find ways to detect this dangerous phenomenon. The results of the first research projects have indicated that there are typical environmental characteristics, such as air temperature and humidity profiles and cloud types, in which intense downward air movements, termed as downburst, occur. The downburst is divided into dry and wet microburst. 

Dry microburst occurs in an environment where a layer of moist air where convective clouds are formed lies above a relatively thick layer (2−3 km) of dry air. Rainfall can be observed at the cloud base; however, it never reaches the Earth’s surface. The falling rain begins to evaporate. This process cools the air, and consequently, it becomes heavier and starts to accelerate towards the ground. When the falling air hits the Earth’s surface, it begins to spread in all directions. Such an air flow, in some cases, may exceed a speed of 70 km/h. Dry microburst is typical for continental and dry climate. It can be detected by lidars. 

Wet microburst is formed in an environment where a layer of dry air is spread above a thick layer of humid air and, at the same time, there are vertically massive convective rain clouds. The most important features of wet microburst include:

- intense downward movements (several units or even tens of m/s);
- areas with high pressure and cold air near the ground below the descending air flow;
- significantly divergent air flow at ground level;
- one or more rotors developed at the edge of the flowing cold air, creating an area of significant horizontal and vertical wind shear, lower pressure and a considerable gradient of several meteorological variables (temperature, humidity, etc.)

Wet microbursts can be detected by radars. Many of the features listed above are dangerous to aviation. Downward flow in the bottom of the downburst may cause a sudden drop in the lift force acting on the aircraft wing. Large wind shear, rotors and turbulence can damage the structure of the aircraft. In addition, many wet microbursts are accompanied by intense rainfall, which can cause a sudden reduction of visibility, which can also lead to accidents. It is believed that the Lion Air Flight 904 accident in Bali in 2013 was due to such conditions [14]. 

The detection of downbursts and related phenomena is difficult for the following reasons:

- The spatial extent of downbursts is small. The dimensions of the areas with their strongest wind often do not exceed a few hundred meters, which are mostly outside of the standard resolution of meteorological radars and much less than the average distance of neighboring automatic weather stations.
- The time span of their existence is short. The time between the beginning of the descending air movement and reaching the ground surface can be estimated as a few minutes. Assuming that the cloud base (the edge of the downburst) is at an altitude of 3 km and the average rate of descent is 10 m/s, the downward flow reaches the Earth’s surface in about 5 min. The total lifespan of a downburst is about 5 to 20 min.
- Segments of the downburst with the potential for the most severe destruction appear only tens or hundreds of meters above the ground, making it difficult to detect by remote detection methods. First, remote observation at low altitudes is problematic both for radars and lidars, particularly in broken terrain. Secondly, radars are seriously handicapped by ground clutters. This effect, on the other hand, does not appear in the case of lidars, due to their narrow and focused beams.

Downbursts can be detected in several ways:

- In the case of a high density of meteorological stations and/or instruments in the area of interest (e.g., airport area), wind gust and other changes in meteorological elements at the surface, typical of downburst, can be at least partially evaluated by computer algorithms.
- The evolution of downbursts can be observed using lidar, especially in dry microbursts.
- The development of wet microbursts can be monitored using radar provided the accompanying clouds are adequately close and the resolution of radar is adequate.

At the same time, the right choice of radar/lidar scanning strategy allows for the detection of both the horizontal and the vertical structure of downburst in a sufficient resolution (at least 250 m or even finer resolution) along the entire radar/lidar beam. The divergent nature of the air flow can be captured in the field of radial Doppler velocities and an algorithm for the automatic detection of the characteristics can be developed [15,16]. 

Obviously, low-level wind shear is not only related to downbursts, but is an excellent representative of seemingly invisible but dangerous meteorological phenomena in aviation, which can be detected in an easier manner by means of lidar technology. Low-level wind shear is commonly associated with the processes in the atmosphere as follows [17]:

- frontal activity;
- thunderstorms;
- temperature inversions;
- surface obstructions.

In the current paper, the above listed processes will be discussed in detail and for the majority of them, an illustrative example will be given and analyzed. 

## 2. Methods and Instruments

### 2.1. Low-Level Wind Shear in Aviation

For aviation purposes, changes of the headwind or tailwind (the longitudinal component of the wind in relation to the orientation of runway) are essential since, generally, the impact of the headwind/tailwind on the aircraft performance during landing or take-off is incomparably more significant than the impact of the crosswind (the lateral component of the wind). Thus, calculations of wind shear over the airport must take into account the orientation of the runways, which means resolving all the shear vectors to the runway headings [18]. 

General recommendations for the estimation of the low-level wind shear originate from the LLWAS system [2]. This system comprises of several anemometers (at least six, at most 32), forming a network of wind measurements over the runways and their vicinity out to 3 nautical miles (NM, ~5.4 km) from the runway edge at the arrival corridor, 2 NM (~3.6 km) from the runway edge at the departure corridor, and 0.5 NM on either side of the extended centerline of the runways, i.e., within the area termed as Areas Noted for Attention (ARENA, [19]). Observations of wind speed and directions are collected from individual anemometers and subsequently, they are processed by the LLWAS algorithm. The divergence in the triangles of three sensors and on the triangle edges between the sensors is calculated and finally, headwind/tailwind gain/loss estimates are produced [18]. Wind shear alerts or microburst alerts are then issued to the pilots on the basis of the magnitude of the headwind gain or loss over a 4 km flight path. An alert “wind shear with loss” is issued when the headwind loss exceeds 7.5 m/s (15 kt) but equals to, or is less than 15 m/s (30 kt). A microburst alert is then issued when a headwind loss of more than 15 m/s (30 kt) over 4 km is observed. A headwind gain of 7.5 m/s (15 kt) or more over 4 km, indicated as “wind shear with gain”, representing areas of convergence (negative divergence), occurs normally ahead of and along a gust front [18]. Wind shear that is detected along a distance exceeding the nominal 4 km is considered of reduced risk since the change in the wind is less abrupt. 

It is important to note that the term “microburst alert” does not necessarily imply that a microburst is literally present at the runway. Instead, microburst alert only suggests that a microburst *may* be present, judged on the basis of the observed wind conditions. 

Wind shear alerts and microburst alerts are delivered to pilots and traffic management specialists through the system Weather System Processor (WSP, [20]) system. The WSP message contains detailed information on the detected phenomena, namely its:

- location (identification of the runway, and whether the phenomena affects its approach or departure corridor),
- character (microburst, wind shear or gust front),
- magnitude (change in the wind speed, loss or gain), and
- the location of the first encounter of the aircraft with the phenomena (whether it can be expected along the runway or 1, 2 or 3 NM from the runway edge).

**Figure 1:**
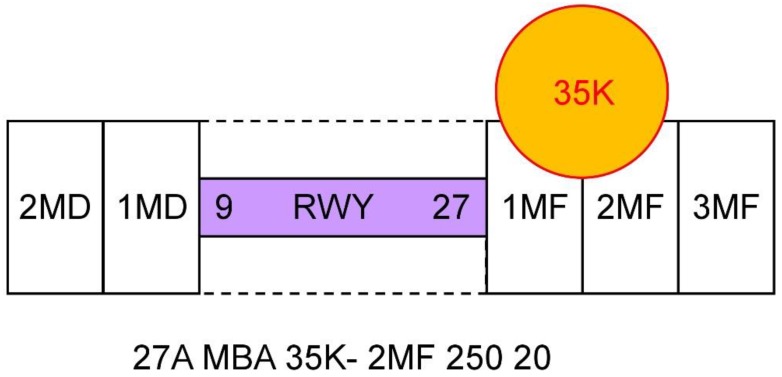
Schematic of a microburst alert in the code of Weather System Processor. The purple rectangle represents the runway (RWY). The rectangles on the right side of the runway denote distance in miles to the final approach (1 MF = 1 nautical miles to the final approach, etc.). Similarly, the rectangles on the left side denote distance from the final departure. The orange circle is a schematic of a microburst at the approach corridor, with the wind speed reaching 35 knots. According to [19].

Figure 1 presents a schematic of a microburst alert. The WSP code 27A MBA 35K – 2MF 250 20 translates as “On approach to runway 27, there is a microburst alert on the approach lane to the runway, and the pilot should anticipate or expect a 35 knot (17.5 m/s) loss of airspeed at approximately 2 miles out on final approach (where it will first encounter the phenomena). Additionally, the surface winds at the airport for landing runway 27 are reported as 250 degrees at 20 knots (10 m/s)” [20]. 

We decided to carry out an analysis of wind shear alerts/microburst alerts according to the ICAO recommendations and the WSP messages in order to estimate the severity of the selected weather events. In the operative practice, the ICAO alerts are issued for the direction of runways. Nevertheless, in the current study, we extended the concept of the ICAO rules in two ways:

(1) We did not focus exclusively on the existing runway directions and their respective corridors. We analyzed each lidar beam instead.
(2) We did not constrain our analysis to a precision of 1 NM, i.e., to the length of the imaginary boxes at the arrival and departure corridors. Instead, we made use of the exact location of an appearance of the wind shear/microburst alerts.

Our algorithm for searching the occurrence of wind shear along the lidar beams is described as follows:

(1) Select a lidar beam and find the furthest gate with a measurement of radial wind speed. Denote this gate as A_1_, with radial wind speed *v*_A1_.
(2) Find the closest gate to A_1_ with a measurement of radial wind speed along the selected lidar beam and towards the lidar. Denote this gate as B_1_, with radial wind speed *v*_B1_.
(3) Let *d* be the distance between A_1_ and B_1_ and Δ*v* = *v*_B1_ − *v*_A1_.
(4) If Δ*v* ≥ 7.5 m/s (15 kt) and *d* ≤ 4 km, then issue *wind shear alert* for the segment A_1_B_1_ and mark it on the lidar snapshot by empty pink circles (see the compositions of lidar snapshots at the selected weather events in the upcoming Sections).
(5) Select a different gate B (B_2_, B_3_, …, B*_N_*) by moving it one, two, …, *N* − 1 gates closer towards the lidar while keeping the gate A_1_ and the lidar beam unchanged.
(6) Repeat steps 3 and 4 *N* − 1 times for different gates B while *d* ≤ 4 km. The final segment with wind shear alert for the gate A_1_ (denoted as A_1_B_WS1_) will consists of the aggregation of the segments with wind shear alerts corresponding to A_1_B_1_, A_1_B_2_, …, A_1_B_N_. Note that all segments marked in step 4) as those with wind shear alert remain also marked after the aggregation of the segments.
(7) Repeat steps 1 to 6 for different gates A (A_2_, A_3_, …) along the selected lidar beam. The final segment with wind shear alert for the selected lidar beam will consists of the union of the segments with wind shear alerts corresponding to A_1_B_WS1_, A_2_B_WS2_, A_3_B_WS3_, …

The algorithm for searching for the microburst alerts is similar, with two minor differences in step 4:

(4) If Δ*v* ≥ 15.0 m/s (30 kt) and *d* ≤ 4 km, then issue microburst alert for the segment A_1_B_1_ and mark it on the lidar snapshot by full pink circles (see the lidar snapshots in the upcoming Sections).

In order to distinguish between the original ICAO algorithm and the one described above, we introduced a convention into the terminology. If the difference of 7.5 m/s (15.0 m/s) in wind speed along the segment of a maximum length of 4 km appears in the ARENA, it will be termed as wind shear alert/microburst alert (abbr. WSA/MBA) in order to indicate similarity with the ICAO algorithm. Otherwise, if the critical wind shear values are exceeded anywhere else beyond the ARENA, we will indicate that the conditions for wind shear alert or microburst alert are fulfilled; briefly the wind shear condition/microburst condition (abbr. WSC/MBC) is met. 

### 2.2. Geographical and Climatological Settings of the Area of Interest

**Figure 2:**
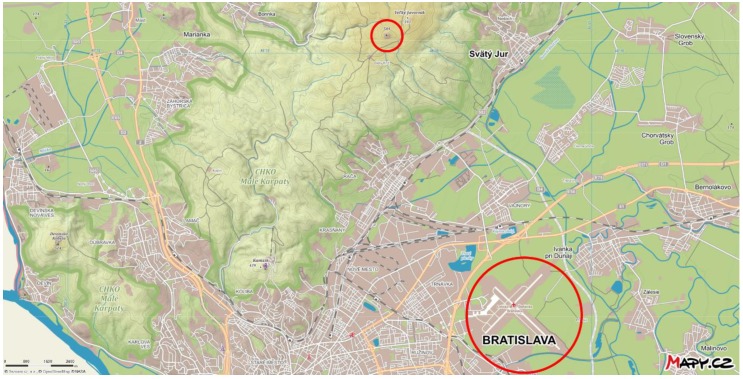
Bratislava Airport (big red circle) and Malý Javorník (the site with the radar of the Slovak Hydrometeorological Institute, small red circle) on the terrain map of the northern parts of Bratislava, including the Protected Landscape Area of the Little Carpathians (CHKO Malé Karpaty). Source: www.mapy.cz.

The climate and especially, the wind conditions in Bratislava are determined by the major geomorphologic units near the city: the Alps, the Carpathians and the Pannonian Basin. From a closer perspective, the Bratislava Airport (112 m above the sea level, abbr. a.s.l.) is mostly influenced by the Little Carpathian Mountain ridge. It is about a 100-km long mountain range extending from Bratislava in a SW to NE direction. The highest peaks of the Little Carpathians exceed 750 m a.s.l. in height; however, peaks near Bratislava reach only up to 400 m over the leeward plain. The Bratislava Airport is located about 10–12 km from the Little Carpathian ridge in the SE direction (Figure 2). 

One of the latest comprehensive analyses of wind climate of Slovakia can be found in the Climate Atlas of Slovakia [21]. The relationship between the topography and the prevailing wind in Slovakia and specifically in Bratislava has been examined by Polčák and Šťastný [22,23]. The average wind speed in the Bratislava area (based on observations from the period 2000–2009) is between 3 and 4 m/s, whereas at similar altitudes in Slovakia, the annual average is 2−3 m/s [21]. This difference can be attributed to the jet effect, stemming from the mutual configuration of the Carpathians and the Alps as well as of the Danube River valley [23]. The average wind speed at the Bratislava Airport is 3.7 m/s. The dominant wind directions are the north-west (NW), west (W) and north (N), while the average wind speed exceeds 4.0 m/s in all of these directions. This fact may be explained by a superposition of the prevailing NW circulation patterns in Central Europe and the topography-induced local jet effect mentioned above. The most frequent wind direction is the NW, with a frequency exceeding 25% of all wind occurrences; north-east (NE) is at the second position with a frequency of 15%–20% [23]. 

### 2.3. Meteorological Instruments at/Near the Target Site

The Bratislava Airport (full name: M. R. Štefánik Airport; IATA: BTS; ICAO: LZIB) is the main international airport in Slovakia, with over 2.2 million of passengers and over 30 thousand landings and take-offs in the year 2018 [24]. It is located in the eastern outskirts of the city, approximately 9 km from the city center. The airport has two perpendicular runways with a concrete surface. The main runway is RWY 13–31 (directions 130° and 310°) with a length (width) of 3190 m (45 m). The second one is RWY 04–22 (directions 40° and 220°), with a length (width) of 2900 m (60 m) [25]. 

Atmospheric processes that are relevant from the perspective of aviation are monitored at the Bratislava Airport by two remote sensing instruments, lidar and radar. Both of them will be introduced in the upcoming subsections. 

#### 2.3.1. Lidar at the Bratislava Airport

Lidar is an acronym of Laser Imaging, Detection and Ranging. Doppler lidar emits pulses of infrared light into the atmosphere and detects rays that are reflected from naturally existing airborne particles of aerosol (dust, water droplets in the clouds or mist, salt crystals, aerosols from biomass burning, etc.). The frequency of the lidar pulses is changed by the wind-induced movement of aerosol. Radial wind speed can then be determined on the basis of the Doppler law by comparing the frequency of the emitted and the reflected light beams. 

The best conditions for lidar measurements are in dusty or obscured atmosphere, since, in these cases, the concentration of aerosols is optimal. A very clean atmosphere with excellent visibility is not favorable for lidars since the concentration of aerosols is not sufficient to reflect the emitted signal. Conversely, low visibility means a too high concentration of aerosols, which results in enhanced absorption of the lidar signal. 

**Figure 3:**
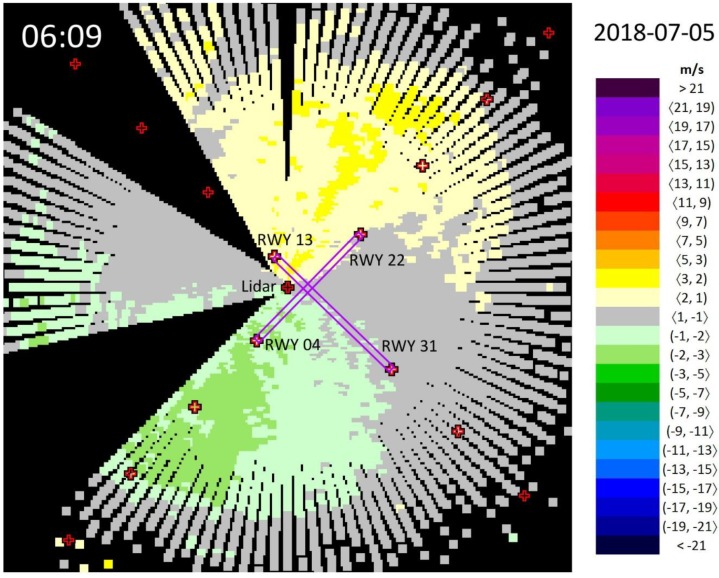
Wind field observed by the Doppler lidar (located at the center of the figure) at the Bratislava Airport at 06:09 UTC on 5 July 2018. Wind blowing towards the lidar is defined as positive. Runways (abbr. RWY) are indicated by the purple color.

The lidar at the Bratislava Airport is located near the cross of the two runways, in the quadrant between 220° and 310°, at a distance of 254.3 m (298.0 m) from the centerline of RWY 13–31 (RWY 04–22) (Figure 3). 

The basic scanning mode of the lidar is a Plan Position Indicator (PPI) regime. In this scanning mode, the optical head of the lidar is set to a constant elevation angle, while azimuth is continuously iterating from 0° to 360° according to the pre-defined angular speed and accumulation time. The result of a single scan is a cone surface, which is displayed in a 2D perspective as a circle. Since the lidar is supposed to provide information on low-level wind shear that pilots may encounter during the phase of landing, the elevation angle is set to 3°, which corresponds with the angle of the glide path of the landing aircraft. 

The most relevant technical parameters of the lidar PPI 3° scans are as follows: the optical head rotates with an angular speed of 3°/s and since the accumulation time is 1.0 s, a full 360° scan is achieved in exactly 2 min. The minimum measured range is 200 m from the lidar, and the display resolution is set as of 100 m. Thus, a single cell of a lidar scan has a width (length) of 3° (100 m). 

As the selected example of lidar snapshots (Figure 3) indicate, there are two blind spots where the lidar beams in the PPI 3° regime are blocked by the surrounding obstacles. The first area lies between the azimuths of 228° and 255° (caused by the airport fire station building), and the second one is delimited by the azimuths of 306° and 327° (caused by the meteorological observatory tower). Note that although the lidar provides some measurements for the azimuths of 306°, 321°, 324° and 327°, they are incomplete due to some blockings and therefore, we decided not to use them in the wind field analysis. 

To each lidar snapshot, a number of fixed reference points (small crosses) was added for a better orientation for the reader (Figure 3). The position of the lidar is marked by the cross in the center of the figures. Furthermore, there are additional 4 × 4 crosses displayed, four along the direction of each runway. In each case, they indicate the end of the runway in the corresponding direction and distances of 1, 2 and 3 NM from the runway edge, respectively, in agreement with the LLWAS methodology [2]. 

Table 1 presents a summary of the distances and altitudes of each reference point relative to the lidar position. Since the maximum range of the lidar is 6−7 km (it depends, however, on the meteorological circumstances, the amount of atmospheric pollutants, etc.), the PPI 3° scan reaches up to elevations of 300–350 m over the surrounding horizontal terrain. Consequently, the 3 NM distance from the end of RWY 31 practically cannot be reached by the lidar beams, thus, it is indicated by brackets in Table 1 and accordingly, the corresponding marker in the south-eastern corner of the lidar snapshots is missing (Figure 3). 

The lidar at the Bratislava Airport has been in operation since 19 June 2018. In the current paper, the lidar database covers the period from the beginning of the measurements until the end of June 2019, i.e., approximately a period of one year. 

#### 2.3.2. Radar at Malý Javorník

One of the four meteorological radars of the Slovak Hydrometeorological Institute (SHMI) is installed at the hill Malý Javorník at an elevation of 584 m a.s.l. and approximately in a horizontal distance of 10.2 km from the lidar at the Bratislava Airport (Figure 2). It is a dual polarization C-band (5 cm wavelength) Doppler weather radar. 

Due to its proximity, the radar can monitor the air space above the airport; however, only from the levels approximately 450−500 m above the airport. The altitude difference between the locations of the two instruments is 472 m. When the radar provides PPI scans at the lowest elevation angle of 0.0°, i.e., in a horizontal direction, the radar beams remain approximately at this elevation above the ARENA. On the other hand, the elevations of the lidar beams corresponding to the furthest points of the ARENA range from about 320 to 420 m (Table 1). Thus, there is no intersection between the lowest elevations reached by the radar and the highest elevations reached by the lidar in the PPI 3° mode. Consequently, the measurements of lidar and radar cannot be compared directly, although they are indeed close to each other. 

In the current study, we made use of two radar products. In case of clear weather, a display of radial wind speed (obtained within a PPI scan under the elevation of 1.5°) is shown. In case of convective processes and related weather phenomena, the CAPPI 1.5 km product was used. Constant Altitude Plan Position Indicator (CAPPI) is a radar display where the altitude above the sea level is kept constant. It is estimated on the basis of several PPI scans that cross the selected altitude. The altitude of 1.5 km is often used to assess the behavior of thunderstorms. 

## 3. Results

### 3.1. Multi-Instrumental Analysis of Selected Weather Events

To analyze the lidar’s and the associated algorithm’s ability to monitor hazardous events in a relatively high spatial and temporal resolution and issue corresponding alerts, we selected weather events that are typical for the target area and are accompanied by considerable low-level wind shear. These meteorological situations demonstrate different circumstances of wind shear genesis: it may appear within a given air mass, on the boundary of two atmospheric layers (during thermodynamic inversion), or as a consequence of fast movements of some formations (cold front, gust front, etc.). Stratification according to typical weather events extends the applicability of the results to wide range of airports in a similar climate.

**Figure 4:**
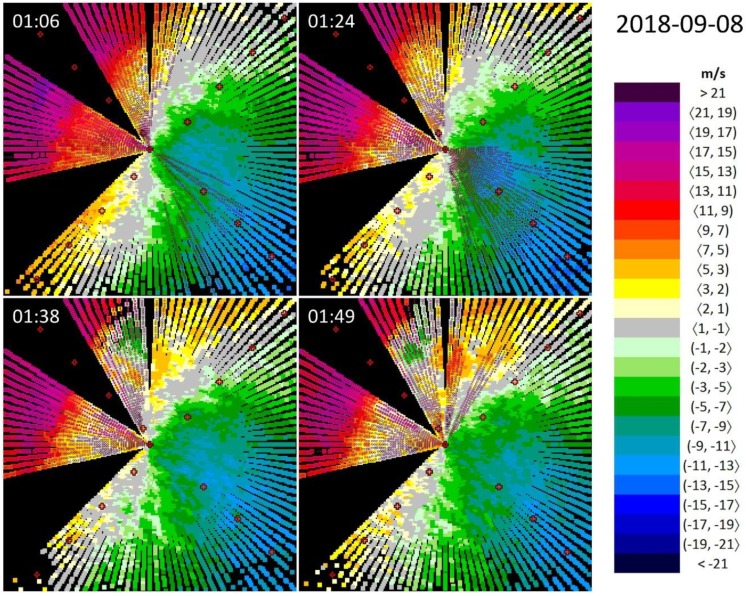
Wind shear in a temporally stable wind field between ~1:00 and ~2:00 AM UTC on 8 September 2018, seen by the lidar at the Bratislava Airport located at the center of each figure. Wind blowing towards the lidar is defined as positive. The empty (full) pink circles indicate wind shear condition (microburst condition) according to the ICAO thresholds.

The selected weather events are presented in terms of a composition of lidar snapshots that are expected to show the most significant features of the temporal (and spatial) evolution of the wind field (e.g., Figure Figure 4) and in terms of tables (Table 2, Table 3 and Table 4), which are meant to summarize further features of the particular weather events (reliability of the measurements, severity of the events, etc.). 

Table 2 shows an analysis of each lidar scan in the selected time period, particularly from the perspective of the individual runways. It presents the number of times when at least one lidar beam with wind shear alert or microburst alert crosses the particular runway with the corresponding arrival corridor. Note that due to the fact that the lidar beams are not completely identical with the runways, for each runway, we tried to restrict ourselves to the 4−5 lidar beams, which (a) show the best coverage with the ARENA and (b) cross the direction of the particular runway at a sufficiently small angle. The only exception is RWY 13 where, due to the blockings by the surrounding terrain objects, only the runway itself is visible for the lidar (and not the extended 3 NM path beyond the runway) and for this reason, we only examined two azimuths with the smallest angles between the lidar beams and RWY 13. 

Table 3 presents statistics of all scanned azimuths, which is a product of the number of azimuths with data for a single lidar scan (108) and the total number of scans from Table 2. The stratification of the azimuths on the basis of beam groups was carried out to check the quality of the lidar measurements. Suppose that in the wind field, there is an odd cell with an unrealistic measurement (noise in data is an inherent part of remote sensing devices like radar and lidar, either due to internal reasons of the device parts and electronics or due to external objects like birds). Without filtering or smoothing the raw lidar data, such a bad cell may lead to false WSA/WSC or MBA/MBC; thus, all the single (isolated) beams with alerts should be double checked and interpreted with caution. It is less probable (although not impossible) that a pair of neighboring alerts along lidar beams that lie next to each other are caused by two neighboring unrealistic cells. On the other hand, one may nearly be certain that a group of azimuths consisting of at least 3 members, each with some alert, cannot be caused by neighboring unrealistic cells. 

The last table (Table 4) expresses the severity of weather events in terms of the largest wind speed differences (wind shear) that occurred during the selected time span and specifies their exact time and location of occurrence. 

Note that the figures in Table 2, Table 3 and Table 4 are the result of a thorough analysis of the numerical outcomes of our algorithm for finding WSA/WSC or MBA/MBC (Section 2.1) and were not determined by visual inspection of the lidar snapshots. The visual inspection was the very last phase of our analysis and it only served to verify the correctness of the figures obtained earlier. 

#### 3.1.1. Event #1—Wind Shear in a Temporally Stable Wind Field 

In the first example (Figure 4), we demonstrate a meteorological situation where low-level wind shear appears within a stable wind field. On 8 September 2018, the wind near the ground at the Bratislava Airport was blowing from the direction of 290° and with a magnitude of 5 m/s. Nevertheless, both the wind direction and the wind speed showed a changing pattern with altitude, as evidenced by the selected lidar snapshots in Figure 4. At 01:06 UTC (Coordinated Universal Time; 03:06 in local time), the wind direction at the altitudes of 300−400 m above the airport is about 310°−320° (indicated by the “S” curvature of the zero isotach), while the wind speed reaches up to the magnitudes of 19 m/s (indicated by an isolated maximum of a violet color in NW direction). Such a change in the wind speed, along the distance of approximately 4−5 km (at the point of 2 NM to the edge of RWY 13) indicates microburst conditions along a number of azimuths between 279° and 303°. 

The location of the significant wind shear remains more or less unchanged as time goes by: microburst conditions can generally be found between 279°−354° and wind shear conditions in a wider sector between 258°−15°. On the basis of the strength of the weather event, one may deduce that similar circumstances might also have been valid in the blind sector around the arrival corridor of RWY 13. Nevertheless, the wind field has no symmetrical arrangement: the wind is less strong on the opposite side (SE) of the lidar. This seems to be a universal feature of the wind at the Bratislava Airport and may be attributed to the proximity of the Little Carpathians in the NW direction. 

Wind shear alerts and microburst alerts based on the ICAO thresholds, as described in Section 2.1, are evaluated in Table 2. It indicates that in the case of RWY 13, alerts (with the dominancy of microburst alerts) appeared in all the 22 selected lidar scans in the period from 01:06 to 01:49 UTC. On the other hand, the lack of the symmetry in the wind field is underpinned by the 14 cases with wind shear alert and no cases with microburst alert in the opposite direction. Table 3 reveals that Event #1 has the highest ratio (~42%) of WSC/MSC to the total number of scanned azimuths among the analyzed four events, which is a sign of a large-scale phenomenon, and the number of questionable alerts (singles or pairs) is also at a relatively low level. The most extreme wind shear observed during this event, as indicated by Table 4, was of a magnitude of 19 m/s. 

The vicinity of the Little Carpathian Mountain range results in another interesting effect. Förchtgott [26] and a number of pilots have reported evidence that in certain circumstances, low-level wind shear appears as a consequence of orography-induced vertical vortices (i.e., in a wider sense, due to the surface obstructions, as mentioned at the end of Section 1.2). It is likely that the lidar snapshots in Figure 4 at 01:38 UTC and 01:49 UTC illustrate such a behavior of the air flow at the leeward side of the mountains. The small green area northward of the lidar that persists for a couple of minutes indicates radial wind speed (‘from the lidar’) in the opposite direction as its vicinity (‘towards the lidar’). To prove this hypothesis, vertical cross-sections of the air space by means of the Range-Height Indicator (RHI) regime or specific lidar settings targeted for the observation of vortices are needed, but unfortunately, for the selected time period we did not have any. Consequently, one of major tasks for the near future is to examine the phenomena of vertical vortices from a closer perspective. 

#### 3.1.2. Event #2—Wind Shear Due to Thermodynamic Inversion

**Figure 5:**
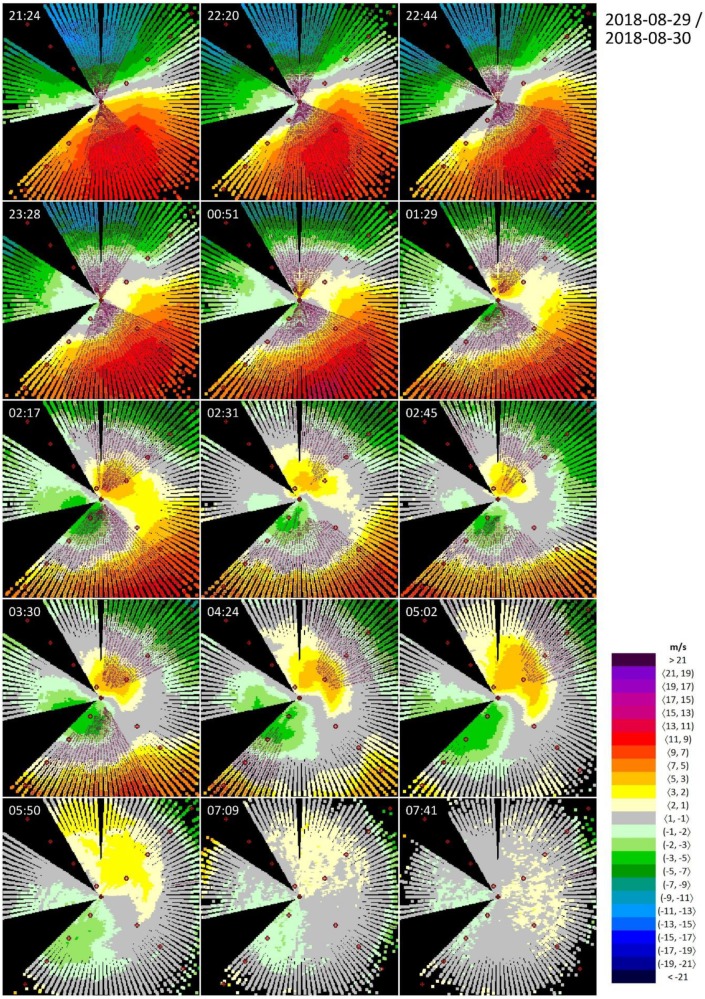
Wind shear on the boundary of two atmospheric layers from ~21 PM UTC on 29 August 2018 until ~8 AM UTC on 30 August 2018, seen by the lidar at the Bratislava Airport, located at the center of each figure. Wind blowing towards the lidar is defined as positive. The empty (full) pink circles indicate wind shear condition (microburst condition) according to the ICAO thresholds.

**Figure 6:**
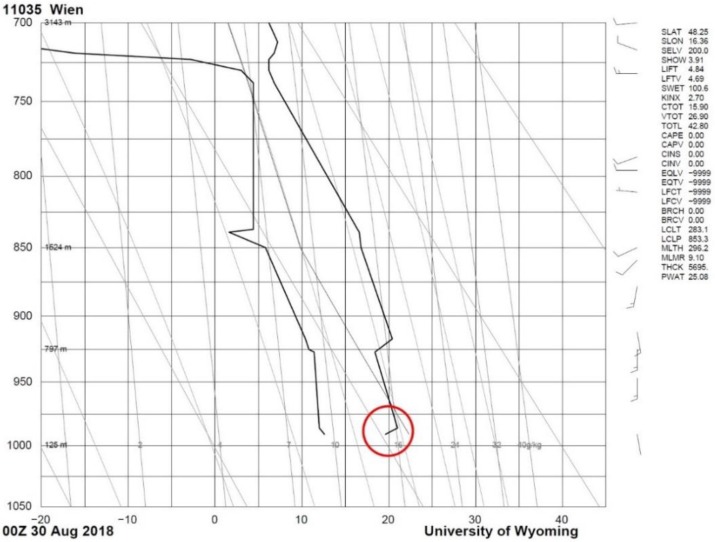
Outcome of the air sounding at midnight on 30 August 2018 at the meteorological station 11035 in Vienna, Austria. The two thick black lines indicate the dry-bulb (right) and the wet-bulb (left) air temperature. The inversion is indicated by the red circle. Source: http://weather.uwyo.edu/upperair/sounding.html.

Vertical discontinuity in the wind field (i.e., wind shear on the boundary of two atmospheric layers) due to thermodynamic inversion is presented as the second example (Figure 5). It spans a longer period of time: from the evening hours on 29 August 2018 until the next morning. A synoptic analysis reveals that the target area was not disturbed by any frontal systems. On the first day, Bratislava was affected by an edge of a diminishing high, and the next day, by a non-significant pressure field. No precipitation was observed during the whole analyzed time period (not shown herein). The thermodynamic inversion is justified by the midnight upper air soundings from Vienna on 29 August 2018, where increasing air temperature can be distinguished from the ground (125 m a.s.l.) up to altitudes of 200−250 m (red circle in Figure 6). Stronger southern wind at higher levels is also indicated by the wind barbs on the right side of Figure 6. Note that Vienna is the closest upper air sounding station to Bratislava, even though it lies at a distance of ~50 km. 

The selected situation (Figure 5) starts on 29 August 2018 after 21:00 UTC. At this time, a wind of a constant SE direction is blowing over the target area, with its speed (2–3 m/s at surface) gradually increasing with elevation and reaching magnitudes up to 13 m/s at heights of 220–320 m over the runways. Later (22:20 UTC), an area of light winds appears near the surface (visible on Figure 5 close to the lidar position); however, the surface wind is nearly in the opposite direction (350° based on the corresponding METAR message) compared to that in the upper levels (still 140°). In the next couple of hours, the wind in the surface layer gets stronger (up to 5 m/s), changes its direction to NNE or NE, and the thickness of this layer increases. At the same time, areas with the highest wind speeds are pushed out to higher elevations and out of the maximum range of the lidar. 

The next day, at the time 03:30 UTC, there are two atmospheric layers over the airport, which are distinctively separated from each other by a boundary that lies approximately in the elevation of 150–180 m over the runways. In these two layers (i.e., above and below the inversion), the radial components of the wind are—by rough estimation—in the opposite direction. In the following hours, the lower layer further increases its thickness, while the upper layer is gradually pushed to higher altitudes. Finally, after 05:50 UTC, the inversion starts falling apart. 

Two types of wind shear can be distinguished in Figure 5 as the thermodynamic inversion evolves. First, wind shear within a stable wind field as a consequence of different wind speeds of the same direction near the runways and in the layers 300−400 m above them, respectively. Wind shear conditions are fulfilled for the azimuths ~310−30°, and symmetrically for ~120−220°. Later, after 01:00 UTC, the wind shear between two atmospheric layers with different (approximately opposite) directions starts dominating and the azimuths with WSC turn slightly clockwise. This behavior results in the fact that all four runways are affected by wind shear alerts approximately in the same manner, each about 110−140 times from the total number of 308 scans selected (Table 2). 

**Figure 7:**
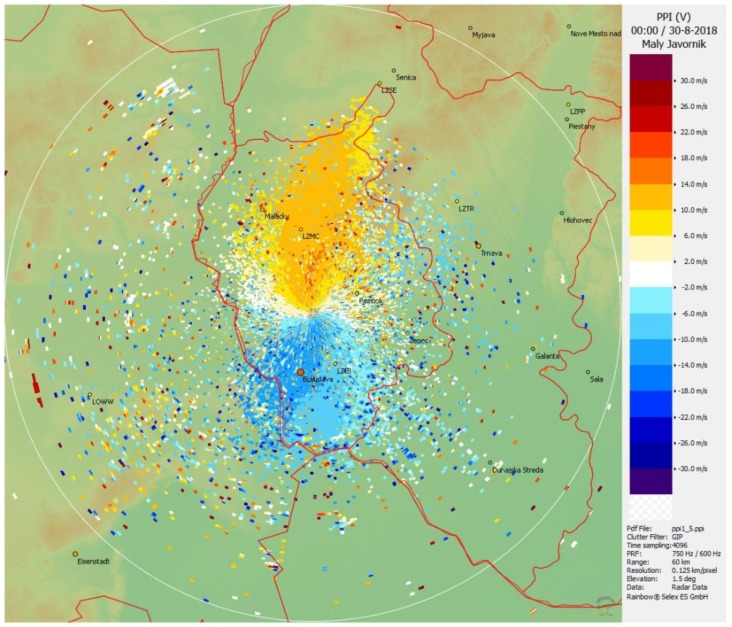
The same event as in Figure 5 but seen by the radar at Malý Javorník and displayed in terms of the radial wind speed in the PPI regime under an elevation angle of 1.5° at 00:00 UTC. Note that in contrast with the lidar, wind blowing towards the radar is defined as negative. The location of the Bratislava Airport is indicated by ‘LZIB’, in the SSE direction from the radar.

The radar snapshot taken at midnight (Figure 7) underpins the findings from the lidar measurements. One can see wind of a speed exceeding 14 m/s blowing from the direction S or SSE in the area of the airport (indicated by ‘LZIB’). After 1:00 AM, the circulation pattern gradually changes: the wind changes its direction to SSW and SW, and at the same time, its speed gradually drops (not shown here). Thus, although radar provides information on the wind field from the areas beyond the maximum range of the lidar beams, it is not able to see the atmosphere close to the surface where the lidar detects wind approximately of an opposite direction. 

Overall, this a moderate and a slowly evolving event where meeting the microburst conditions are not expected. Table 3, however, indicates that there are 28 occurrences of isolated (single) microburst conditions. 

We inspected these cases one by one thoroughly and concluded that all cases of MBC were caused by cells with invalid measurements that predominantly occurred near the maximum range of the lidar. From the perspective of automated algorithm, our quality check comparing neighboring beams filtered out these cases. Therefore, the magnitude of the maximum wind shear (13.7 m/s) in Table 4 is consistent with the above-described features of the selected weather event. 

The two weather events discussed so far were related to clear air situations. In contrast, in the next two subsections, we will present events that were accompanied with visible and significant phenomena, such as clouds of a high vertical extent, heavy precipitation and rapidly changing wind patterns. 

#### 3.1.3. Event #3—Wind Shear at the Passage of a Cold Front

The selected weather event occurred on 5 July 2018. The day before the target day, the sea level pressure over Central Europe was insignificant, with no influence of relevant highs or lows. The air was predominantly warm and moist. A cold front from the west was gradually approaching towards Central Europe, with an enhanced potential for thunderstorms. The weather forecast of the Slovak Hydrometeorological Institute issued on 4 July 2018 expected that the first local thunderstorms would appear on 5 July 2018 in the western and northwestern regions of Slovakia (including Bratislava). The thunderstorm activity was supposed to intensify in the evening hours and to move slowly from west to east. On 5 July 2018, SHMI issued alerts on the expected thunderstorm activity where the first warning level (on a scale where the maximum level is 3) was indicated for the western and northwestern parts of the country. 

**Figure 8:**
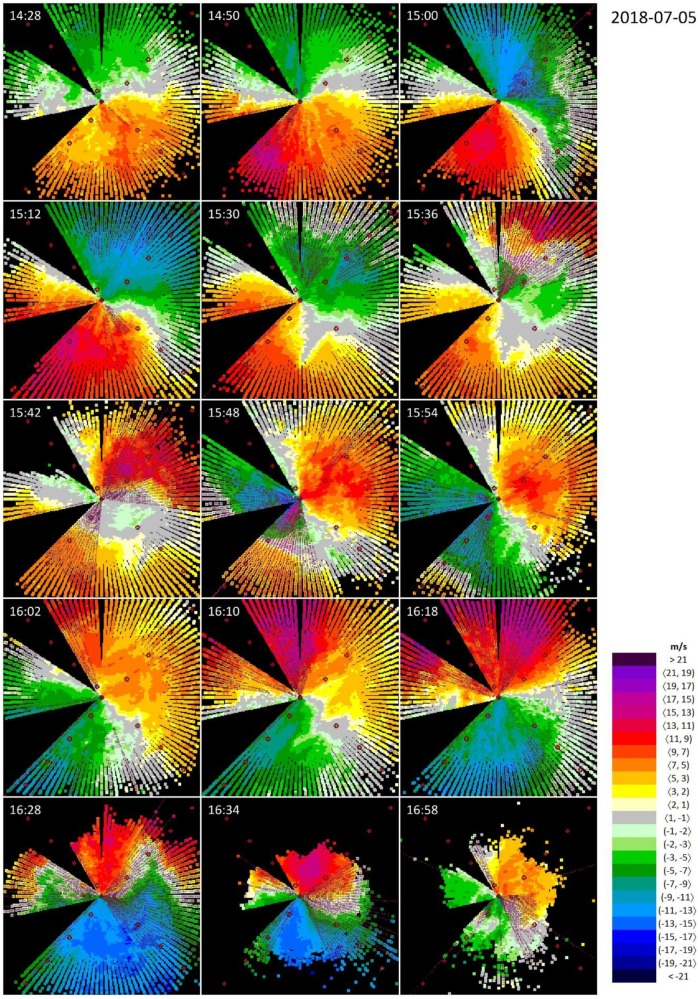
Wind shear at the passage of a cold front from 14:28 UTC until ~17:00 UTC on 5 July 2018, seen by the lidar at the Bratislava Airport, located at the center of each figure. Wind blowing towards the lidar is defined as positive. The empty (full) pink circles indicate wind shear condition (microburst condition), according to the ICAO thresholds.

**Figure 9:**
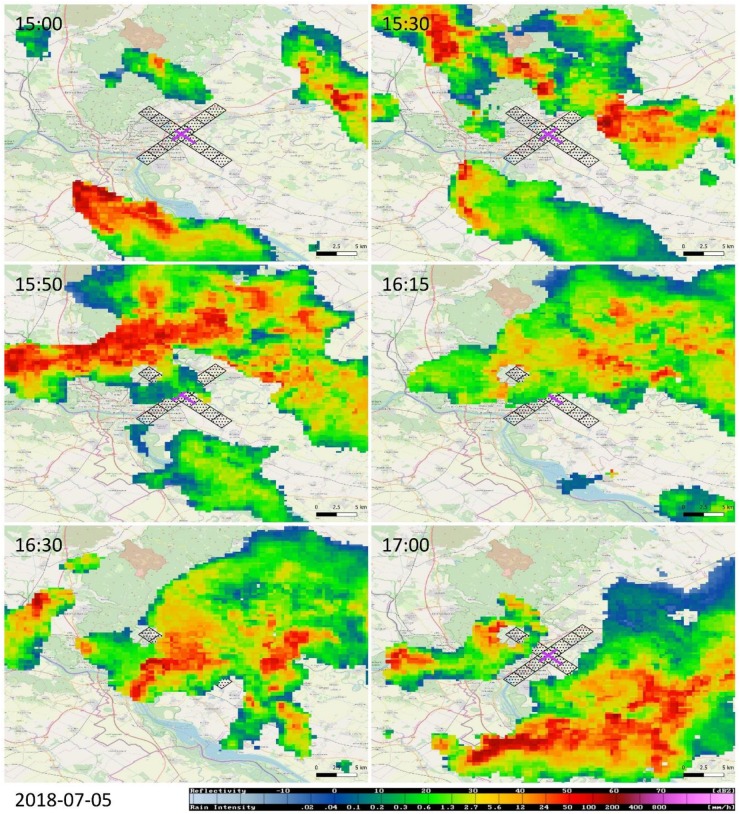
The same weather event as in Figure 8, but seen by the radar at Malý Javorník and displayed in terms of the CAPPI 1.5 km product. The Bratislava Airport is highlighted by the purple cross, while the corresponding ARENA is indicated by the black-dotted rectangles.

The evolution of the selected weather event is documented in Figure 8 and Figure 9. It all starts at about 14:30 UTC on 5 July 2018, when stable wind blows approximately from the direction of 160° with a maximum radial wind speed of 9 m/s, and neither the wind direction nor the wind speed change with altitude (Figure 8). Around 15:00 UTC, the wind field is disturbed by an inbreak of stronger wind from ~210°, with a maximum wind speed of 15 m/s (Figure 8). The increased wind speed is due to an intense thunderstorm cell approximately 15 km SE from the edge of RWY 04 (Figure 9, 15:00 UTC). The new status of the wind field, however, remains stable only for about 20 min. 

After 15:30 UTC, a new formation with strong radial wind appears in NE and gradually moves towards the lidar (Figure 8). Radar imagery (Figure 9, 15:30 UTC) reveals a thunderstorm cell developing NE of Bratislava and the strong wind detected by lidar a couple of minutes later (Figure 8, 15:36 UTC) represents the associated gust front moving towards the airport. In some moments, at the mid-elevations (150−200 m above the ground, see Table 1), the wind blows towards the lidar both form the NE (due to gust front movement) and SW (due to the prevailing flow in the area) directions—for instance, at the snapshot taken at 15:42 UTC (Figure 8). 

The new stable situation (Figure 8, around 15:50 UTC) can be characterized by wind from ~40°, with a maximum wind speed of 13 m/s. This wind field, again, is disturbed by intense air flow, which is formed on the front side of the thunderstorm that approaches the lidar from the north (Figure 9, 15:50 UTC). At 16:18 UTC, the maximum wind speed reaches up to 19 m/s (Figure 8). This last disturbance of the wind field, however, is accompanied by rainfall: it is underpinned (i) by the attenuation of the laser beams of the lidar (around 16:30 UTC, Figure 8) and (ii) by the thunderstorm cell crossing over the runways (16:30 UTC, Figure 9). As evidenced at 16:58 UTC (Figure 8), the wind finally calms down as the storm cell leaves the area of the airport (Figure 9, 17:00 UTC). 

Due to the first inbreak of strong wind from the NE direction, RWY 04 and RWY 22 are the most affected ones by wind shear alerts or microburst alerts (Table 2). In comparison with the weather events analyzed in the previous sections, Event #3 has the most turbulent character, with a multitude of sudden changes in the wind field. This fact is underpinned in Table 3, where both the highest percentage of the potentially unreliable occurrences of WSC/MBC (‘single’ beams with alerts) and the lowest percentage of the WSCs/MBCs grouped in larger groups, respectively, are related to Event #3. Finally, Table 4 indicates that the passage of a cold front was the second most severe among the analyzed ones, with the maximum wind shear at some points exceeding 20 m/s. 

#### 3.1.4. Event #4—Wind Shear at the Passage of a Gust Front

The last example demonstrates an exceptionally intense weather event that took place during a very short period (practically during an interval of 20 min) in the evening hours on 2 September 2018. The first days of September 2018 showed up with a very turbulent weather in Central Europe, especially in Bratislava. As a result of a cold front, the capital city of Slovakia had to face several waves of intense rain showers in the evening hours on 1 September 2018. Due to the severe weather, a number of streets and roads remained under water and plenty of cellars and underpasses were flooded. Forecast issued by SMHI on 1 September 2018 expected a similar behavior of the weather on the following day with thunderstorms and rain showers and issued a warning of the highest (third) level on possible extreme precipitation amounts, exceeding 90 mm, and wind gusts reaching 16−20 m/s for Bratislava and its close vicinity.

**Figure 10:**
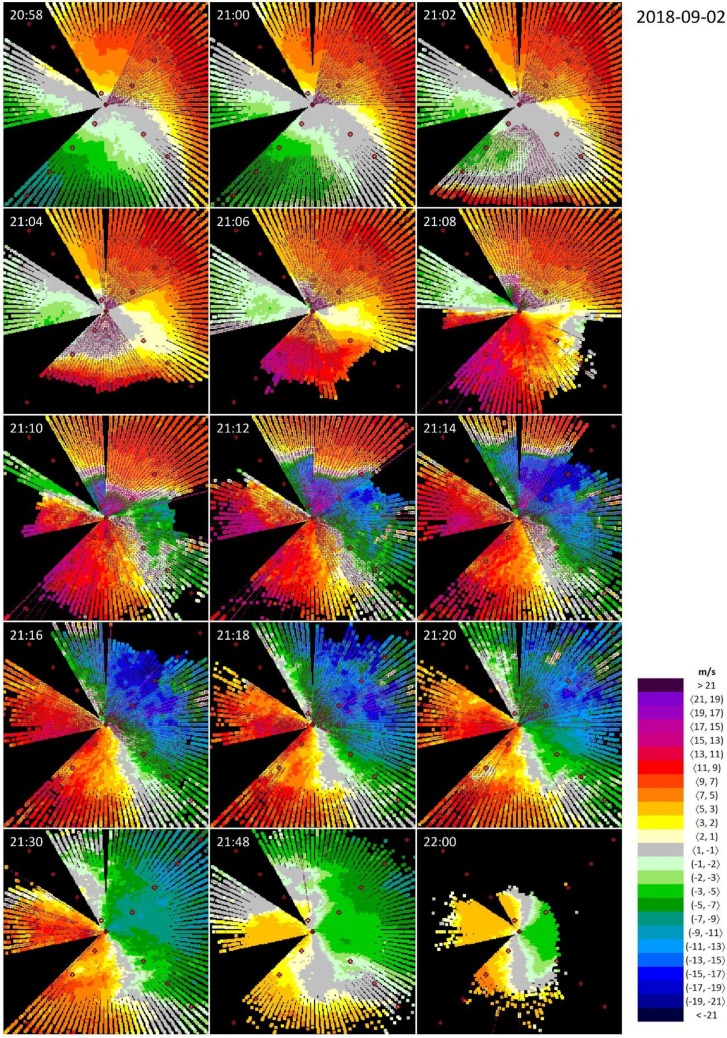
Wind shear at the passage of a gust front from 20:58 UTC to 22:00 UTC on 2 September 2018, seen by the lidar at the Bratislava Airport located at the center of each figure. Wind blowing towards the lidar is defined as positive. The empty (full) pink circles indicate wind shear condition (microburst condition) according to the ICAO thresholds.

Figure 10 shows the evolution of the weather event where the precursors of the low-level wind shear may be classified as thunderstorm-related (see the end of Section 1.2). Due to the intensity of the atmospheric processes, we did not skip any of the lidar snapshot from the first 22 min of its life cycle. At the beginning, at 21:00 UTC, the anemometer near the ground indicated weak and variable wind, with a speed of 2 m/s. The lidar snapshot, however, reveals that there is significant air motion above: the wind blows from NE and the wind speed reaches magnitudes up to 11 m/s at elevations of about 400 m above the ground. This yields wind shear conditions along a number of azimuths in the NE to E, and in the S to SW directions, respectively. 

**Figure 11:**
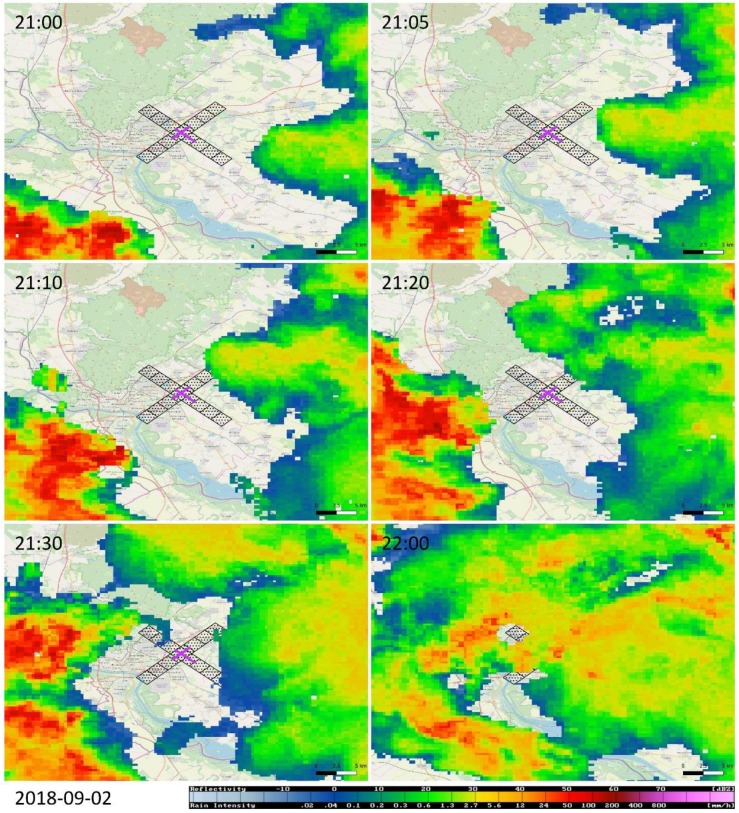
The same weather event as in Figure 10, but seen by the radar at Malý Javorník and displayed in terms of the CAPPI 1.5 km product. The Bratislava Airport is highlighted by the purple cross, while the corresponding ARENA is indicated by the black-dotted rectangles.

At 21:02 UTC, a gust front appears SW from the lidar and moves very fast northward (Figure 10). It origins from a thunderstorm cell in the SW direction approximately 10−15 km from the edge of RWY 04, which can be seen the radar snapshot a few minutes earlier, at 21:00 UTC (Figure 11). Thus, the combination of the lidar and the radar views reveal a typical weather situation that is invisible but highly dangerous for an aircraft. The wind speed in the gust front may exceed 17 m/s (Figure 10, 21:06 UTC). 

As the gust front progresses northward, its front side passes by the lidar at about 21:08 UTC, and reaches the point of 2 NM to the edge of RWY 22 at about 21:14 UTC. Thus, the speed of progressing of the gust front can be estimated as of ~5 km in 8 min, i.e., ~35–40 km/h. When examining the movement of the thunderstorm cell on the CAPPI products, we can arrive at a similar conclusion, since the core of the cell progresses in a NW direction about 15 km in a 30-minute period (Figure 11, 21:00–21:30 UTC). During the period of 21:08 and 21:14 UTC, a wide sector with microburst condition can be distinguished in the N−NE directions for a couple of minutes but, as evidenced by Figure 11, there is still no rainfall in the ARENA. 

As the gust front passes by, a new stable situation takes over (at about 21:30 UTC) whereby the wind blows from SW with wind speed at a magnitude of 11 m/s. At about 22:00 UTC, the wind calms down, but one can distinguish a slight turn of the wind direction from W to SW in its vertical profile (Figure 10) and at the same time, the whole ARENA is hit by intense rainfall (Figure 11). 

This gust front is the most severe one among the four weather events discussed. Table 4 indicates that the largest wind shear observed during this event was as of 27.0 m/s. It can be clearly seen at the snapshot at 21:14 UTC that this is the result of the collision between the stable wind field blowing from N (with a radial wind speed of up to ~11 m/s) and the gust front that moves fast northward with wind blowing from S (with radial wind speed up to ~17 m/s). 

### 3.2. Monitoring the Aerosol Content

In the context of lidar, it is not only the low-level wind shear that influences human health. Through the measurement of the height of the boundary layer and the aerosol content in it, lidar may provide further valuable information on the conditions that directly affect the human respiration. 

**Figure 12:**
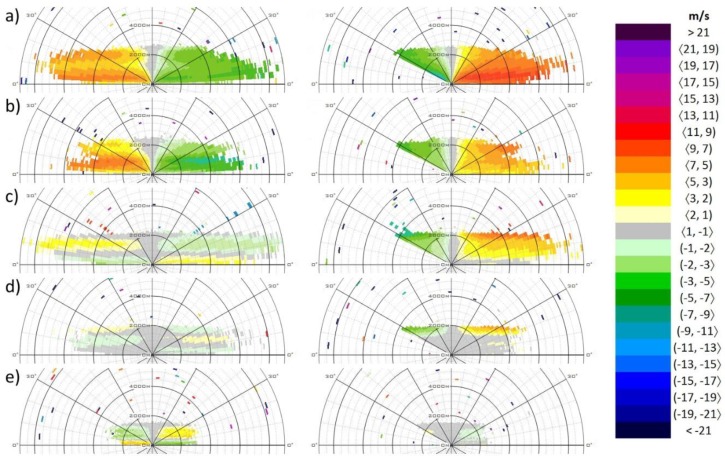
Vertical cross-sections obtained by the RHI scanning regime of lidar. Left column: RHI scans in the orientation of 40° and 220°. Right column: RHI scans in the orientation of 130° and 310°. (**a**) 19 September 2019, 20:17 UTC; (**b**) 20 September 2019, 02:17 UTC; (**c**) 20 September 2019, 20:23 UTC; (**d**) 21 September 2019, 02:15 UTC; (**e**) 21 September 2019, 06:20 UTC. Wind blowing towards the lidar is defined as positive.

We present a composition of vertical cross-sections of the atmosphere (Figure 12), obtained by means of the RHI regime of the lidar. In this mode, the azimuth of the lidar is set to a fixed value, while the elevation angle changes from 0° to 180°, i.e., to 0° at the opposite side. In the case of the lidar at the Bratislava Airport, the orientation of the RHI scans are identical with the orientation of the runways. Figure 12 shows a selection of a variety of meteorological situations that were observed during a 3-day period in September 2019. Pairs of the RHI scans corresponding to the orientation of 4−22 (left column of Figure 12), and 13−31 (right column of Figure 12) were obtained with about a 1-minute difference. 

Figure 12a (19 September 2019, 20:17 UTC) is a typical example of the daytime boundary layer: aerosols are dispersed towards higher altitudes (~2700 m) due to the daytime turbulence. Figure 12b (20 September 2019, 02:17 UTC) is a follow-up situation to the previous one: turbulence is stopped during the nighttime but the aerosol particles are still dispersed within the residual layer. Figure 12c (20 September 2019, 22:23 UTC) presents a nice example of a thermodynamic inversion where the radial wind speed in the bottom layer reaching up to elevations of 700 m is in the opposite direction compared with that in the layer above. Approximately four hours later, the situation is slightly different: the RHI scan in directions 4−22 (Figure 12d) indicates a further subdivision of the boundary layer into three distinctive parts. Finally, Figure 12e (21 September 2019, 06:20 UTC) indicates that the nocturnal boundary layer under the capping inversion is only 500 m deep 

The composition of RHI snapshot in Figure 12 illustrates that the boundary layer is a dynamically varying part of the lower troposphere that keeps changing both its extent and content. Beyond being useful in aviation, the RHI scans also provide valuable information on the aerosol content not only for close neighborhood of the airport, but also for the wider area of the urban agglomeration. The height of the boundary layer is a significant parameter in modelling the dispersion of pollutants. When exceeding 1.5−2 km, it indicates favorable conditions for the dispersion of the aerosols, i.e., low concentration of the pollutants near to the ground. Small extent of the boundary layer, on the other hand, imply a higher concentration of pollutants and worse conditions for the respiration of human beings. 

## 4. Discussion

In the current study, we tried to adopt the ICAO algorithm for issuing wind shear and microburst alerts and extend it in a way that naturally emerges as a consequence of the lidar-based technique of wind shear detection. The performance of our algorithm was verified by means of selected weather events with an occurrence of low-level wind shear. Some aspects of our methodology are discussed in more detail below:

### 4.1. Spatial Consistency 

Our algorithm, which estimates the occurrence of wind shear or microburst conditions in the entire range of the lidar beams, gives useful feedback on the spatial consistency of the particular weather event. It is expected that a very strong wind shear is manifested by a relatively wide sector of lidar beams with microburst conditions, which is embedded within an even wider sector of beams with wind shear conditions. The sudden occurrence of an isolated lidar beam (or a pair of them) with a fulfilled microburst condition, on the other hand, should indicate the presence of some unrealistic lidar measurement(s). 

### 4.2. Wind Shear Alerts on the Runways 

The statistics presented in Table 2 report on the number of cases when conditions are fulfilled to issue a wind shear alert or a microburst alert on the particular runway and the corresponding arrival corridor. We focused on the 4−5 lidar beams that are closest to the given runway in order to cover the ARENA as completely as possible. Nevertheless, we are aware of the fact that our approach is not perfect. There may be cases, for instance, when wind shear alert is issued for a given runway on the basis of the fact that the conditions for issuing the alert are met at the very end of the particular lidar beam that is beyond the ARENA. Therefore, our algorithm needs minor refinement, which would derive the information on wind shear or microburst alert only on the basis of the segment of each lidar beam that cross the area of the ARENA. Moreover, there is potential for a much more significant improvement: to introduce specialized glide path scans which would be targeted on the close vicinity of the aircraft’s glide path, both in terms of a narrow spectrum of azimuths and elevations, similarly to the concept of the GLYGA algorithm [3]. 

### 4.3. Rapidly Changing Conditions 

The series of the lidar snapshots in Figure 8 presents an evolution of a weather event when the dominant wind conditions changed significantly three times during the time period of 2.5 hours. At some moments, the changes in the wind field took place so fast that the lidar was not able to display them correctly. This can be illustrated by a seeming discontinuity in the wind field in the northern direction on the snapshot at 15:36 UTC. This is not the failure of the lidar: it starts scanning in the northern direction and after a 360-degree rotation that lasts for 2 min, the lidar beam returns to its starting position where, however, the meteorological conditions considerably differ from those observed 2 min earlier. A similar discontinuity due to rapid changes in the wind field can be distinguished in Figure 10. One cannot completely get rid of the observed discontinuities; however, one can at least reduce them by changing the scanning strategy, for instance, by setting up a higher rotation speed of the lidar optics. On the other hand, such a benefit may be outweighed by a possible degradation in the quality (worse resolution) of the lidar measurement. 

### 4.4. Lidar in the Center 

The search for the occurrence of wind shear (or microburst) conditions is always carried out along the lidar beams where the lidar itself represents the endpoint of the searching procedure. One could, in principle, obtain a more comprehensive picture of the wind shear if, together with the particular lidar beam, the one in the opposite direction was also analyzed. In practice, however, this is not an ideal solution since there is a 1 min difference in the measurement along these two azimuths, and as discussed above, rapid changes may take place in the wind field. The gust front demonstrated in Figure 10 is an excellent illustration of this problem. We know that the wind field is continuous and there is significant wind shear (with microburst conditions) in the target area even at 21:04 and 21:06 UTC, but we cannot see it since the algorithm breaks the wind field into two parts and analyzes separately what happens northward and southward to the lidar, respectively. As soon as the front side of the gust front appears at the northern side of the lidar (from 21:08 UTC onward), this drawback of the algorithm diminishes and the microburst conditions become apparent. 

### 4.5. Seasonal Differences 

The lidar scanning strategy should, in a long-term plan, also consider the seasonal differences in the behavior of the weather in Central Europe. During the warm half-year (approximately from April to September), weather events with a convective character dominate, so more stress should be given to the PPI scanning regime. On the other hand, the cold half-year (October−March) can be characterized by less turbulent weather, with frequent occurrences of thermodynamic inversions, fogs and stratiform type of precipitation, which should be reflected by giving higher priority to the RHI regimes in the scanning strategy. 

### 4.6. Scanning Strategy 

As discussed above, a number of improvements may be achieved by setting up a more targeted scanning strategy. Nonetheless, this is a complex task since one must take into consideration different aspects, from the technical parameters of the lidar to the features of the weather/climate at the target area and the demands from the future end users. Improvement in one direction may lead to worse lidar performance in another; therefore, it is not a straightforward optimization problem. 

## 5. Conclusions

The most important conclusions of the current work may be summarized as follows:

- Lidar is able to monitor the evolution of weather events at a high spatial and temporal resolution. This is a very useful finding both from the perspective of aviation and air pollution modelling, since no similar opportunities have been available to date in the broader area of Central Europe.
- In the climatic conditions of Bratislava, medium-type lidar (Leosphere WindCube 200S) can usually “see” up to 6−7 km approximately horizontally (more precisely, along the glide path under the elevation of 3°) and 1−2.5 km vertically in the RHI regime. This finding may be extended to areas with similar climate and aerosol contents, i.e., to regions lying on the boundary of the maritime and the continental climatic regimes.
- Lidar does not replace radar. Lidar beams are more attenuated by rainfall (see Events #3 and #4) than those of the radar, therefore, in showery rain, radar can “see” further than lidar. The Bratislava Airport, however, has a specific (unfortunate) feature: due to the orographic settings (where radar is elevated and lidar is installed on the plain), the radar cannot see the airport. On the other hand, radar observations provide useful information on the radial wind speed at the higher levels of the boundary layer but not for the final phases of an aircraft’s landing. Thus, the lidar and the radar in Bratislava complement each other not only from the perspective of whether they are more suited to monitor dry or wet weather events but also in the aspect of the topographical configuration of the target area.
- Analysis of low-level wind shear and issuing wind shear alerts (microburst alerts) that meet the ICAO requirements is possible on the basis of lidar observations.
- Automated wind shear alerts may suffer from false alarms generated by noise in the data. Therefore, quality check is inevitable, as was shown by a cross-check by means of the neighboring beams with alerts.
- From the perspective of aeronautical safety, it is important to stress that many of the dangerous weather situations are “invisible” (demonstrated by the example of Events #1 and #2)—the wind shears occur in clear and seemingly peaceful atmosphere at a high pressure. Without lidar capable of scanning such situations, the pilot standardly receives measurements of the surface wind only, which are directly opposite to the winds aloft, encountered shortly before landing and after take-off. Such cases are often reported by pilots using Bratislava Airport [personal communication].
- For civil protection, the protection of human lives and health and air pollution modelers, it is crucial information that any chemical substances or smoke from potential accidents or pollutant and aerosols from regular sources, will be trapped in the lower 700 m of the atmosphere and may potentially reach dangerous concentrations. Such measurement is hardly obtainable otherwise, as Bratislava does not operate upper air sounding station (which is the usual state of towns with their population over half a million).

There are different pathways to take to continue our research within the framework of the current scientific project. First, the expert-based synoptical analysis of further meteorological situations such as stable vertical vortices from the nearby mountains will be presented in a similar manner. Secondly, computer-based automated algorithms for gust front and/or downburst recognition will be adopted and developed to enhance the flight safety and protect human lives and health in connection with the aviation activities in the specific conditions. 

## Figures and Tables

**Table 1:** Distances and altitudes of the reference points (marked as crosses) relative to the lidar position in the lidar snapshots. Abbreviations: RWY = Runway, 0 NM = runway end, *d*—horizontal distance from the lidar, *H*—altitude over the surrounding terrain.

Distance	0 NM	0 NM	1 NM	1 NM	2 NM	2 NM	3 NM	3 NM
RWY	*d* [m]	*H* [m]	*d* [m]	*H* [m]	*d* [m]	*H* [m]	*d* [m]	*H* [m]
22	1770	93	3570	187	5370	281	7170	376
31	2590	136	4390	230	6190	324	(7990)	(419)
04	1200	63	3000	157	4800	252	6600	346
13	670	35	2470	129	4270	224	6070	318

**Table 2:** Evaluation of the selected weather events (#1−#4) from the perspective of lidar scans: how many times the given runway and its corresponding arrival corridor meet with at least one lidar beam marked with wind shear alert (WSA; 7.5 m/s ≤ Δ*v* ≤ 15.0 m/s, i.e., empty pink circles) or only microburst alert (MBA; Δ*v* ≥ 15.0 m/s, i.e., full pink circles).

Runway	Category	Event #1	Event #2	Event #3	Event #4
	Start date	2018-09-08	2018-08-29	2018-07-05	2018-09-02
	# of scans	22	308	76	32
22	WSA	0	118	28	14
	MBA	0	0	5	3
31	WSA	14	139	10	5
	MBA	0	0	0	0
04	WSA	0	130	24	15
	MBA	0	0	2	1
13 *	WSA	6	112	18	5
	MBA	16	0	0	2

* In the case of Runway 13, only its cross with the lidar beams at the azimuths of 330° and 333° is counted.

**Table 3:** Evaluation of the selected weather events (#1−#4) from the perspective of all the scanned azimuths (i.e., all lidar beams): number of azimuths with fulfilled conditions for wind shear (WSC; 7.5 m/s ≤ Δ*v* ≤ 15.0 m/s, i.e., empty pink circles) or for microburst (MBC; Δ*v* ≥ 15.0 m/s, i.e., full pink circles), which are isolated (Single), form pairs next to each other (Double) or form groups with at least three members next to each other (3+).

Group	Category	Event #1	Event #2	Event #3	Event #4
	Start date	2018-09-08	2018-08-29	2018-07-05	2018-09-02
	# of azimuths	2244	31416	7752	3264
	# of WSC/MBC conditions	933 (41.6%)	11513 (36.6%)	1976 (25.5%)	1106 (33.8%)
Single	WSC	13 (1.4%)	286 (2.5%)	244 (12.3%)	68 (6.1%)
	MBC	17 (1.8%)	28 (0.2%)	28 (1.4%)	15 (1.4%)
Double	WSC	4 (0.4%)	122 (1.1%)	118 (6.0%)	50 (4.5%)
	MBC	16 (1.7%)	0 (0%)	12 (0.6%)	10 (0.9%)
3+	WSC	680 (72.9%)	11077 (96.2%)	1500 (75.9%)	856 (77.4%)
	MBC	203 (21.8%)	0 (0%)	74 (3.7%)	107 (9.7%)

**Table 4:** Evaluation of the selected weather events (#1−#4) in the aspect of wind shear severity: the maximum difference in wind speed (Max. Δ*v*) along a distance of 4 km or less, with the exact time and location of their occurrence. WSC = wind shear condition, MBC = microburst condition.

Category	Event #1	Event #2	Event #3	Event #4
Max. Δ*v* [m/s]	19.0	13.7	22.0	27.0
Date	2018-09-08	2018-08-30	2018-07-05	2018-09-02
Time	1:38	0:53	15:36	21:14
Azimuth [°]	351	162	33	36
Distance A [m]	4300	1100	1500	2100
Distance B [m]	8200	5100	5300	6100
Δ*d* [m]	3900	4000	3800	4000
Condition	MBC	WSC	MBC	MBC
